# Nasal kaposiform hemangioendothelioma: a rare disease in a rare location—a review article with a case report

**DOI:** 10.3389/fsurg.2026.1728160

**Published:** 2026-05-13

**Authors:** Feras Alkholaiwi, Ji Yun Choi

**Affiliations:** 1 Department of Otorhinolaryngology-Head and Neck Surgery, College of Medicine, Imam Mohammad Ibn Saud Islamic University (IMSIU), Riyadh, Saudi Arabia; 2 Department of Otorhinolaryngology-Head and Neck Surgery, Chosun University College of Medicine, Gwangju, Republic of Korea

**Keywords:** endothelioma, hemangioma, kaposiform hemangioendothelioma, nasal, sinonasal

## Abstract

Kaposiform hemangioendothelioma is a rare vascular tumor of endothelial origin that occurs during the first decade of life. It is locally aggressive, with a tendency for local invasion and spread to regional lymph nodes. It presents as tender, expanding plaques, nodules, papules, or telangiectasias and is most commonly located in the retroperitoneum and skin; however, involvement of the head and neck region is uncommon. Herein, we report an unusual case of kaposiform hemangioendothelioma in a 59-year-old woman who presented with a several-month history of right-sided nasal obstruction and epistaxis. Contrast-enhanced computed tomography revealed an enhancing mass originating from the right inferior turbinate. The patient underwent endoscopic excision of the endonasal mass with any postoperative complications.

## Introduction

Kaposiform hemangioendothelioma (KHE) is an infrequent tumor of endothelial origin that occurs predominantly in infancy and early childhood (1). It was first described in 1971 as “hemangioma with Kaposi's sarcoma-like features” and has since been referred to by other synonyms, such as Kaposi-like infantile “hemangioendothelioma” and “hemangioendothelioma,” were also used to describe this tumor (2, 3). The term “Kaposiform hemangioendothelioma” was first coined by Zuckerberg in 1993 (4). The tumor is locally aggressive, with a tendency for local recurrence and spread to regional lymph nodes. The World Health Organization classifies this tumor as having intermediate malignant potential (5). It presents as tender, expanding plaques, nodules, papules, or telangiectasias and is most commonly located in the retroperitoneum and skin. Its occurrence in the head and neck region is relatively rare.

Here, we report an unusual case of kaposiform hemangioendothelioma arising in a rare location. To the best of our knowledge, only a few cases of nasal tumors have been reported in the literature.

## Case report

A 59-year-old woman presented with a several-month history of right-sided nasal obstruction and epistaxis. The patient denied experiencing any other nasal symptoms and had no recent history of trauma or surgery. A comprehensive otorhinolaryngologic and head and neck examination was performed, including nasal endoscopy.

**Figure 1:**
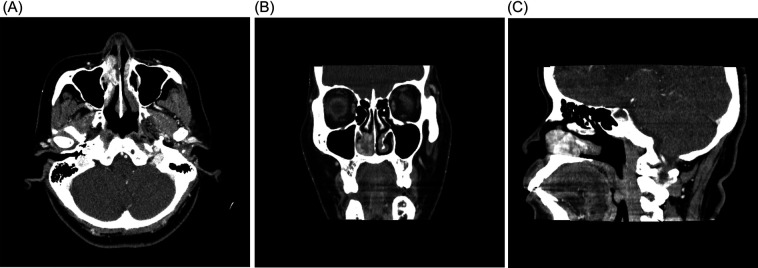
Preoperative imaging studies. Contrast-enhanced CT **(A)** axial, **(B)** coronal, and **(C)** sagittal views showed an enhancing mass originating from the right inferior turbinate, extending medially toward the nasal septum and posteriorly to the middle of the nasal cavity at the level of the inferior meatus.

Endoscopic findings revealed a right-sided nasal mass originating from the right inferior turbinate and obstructing the nasal passage. The mass measured approximately 3 cm × 2 cm, was dark red in color, soft on palpation, and showed no evidence of ulceration. Contrast-enhanced computed tomography (CT) (Figure 1) revealed an enhancing mass originating from the right inferior turbinate and occupying the inferior meatus and nasal passage. Preoperative blood investigations, including complete blood count, hemoglobin level, partial thromboplastin time, prothrombin time, and international normalized ratio , were all within normal limits.

**Figure 2:**
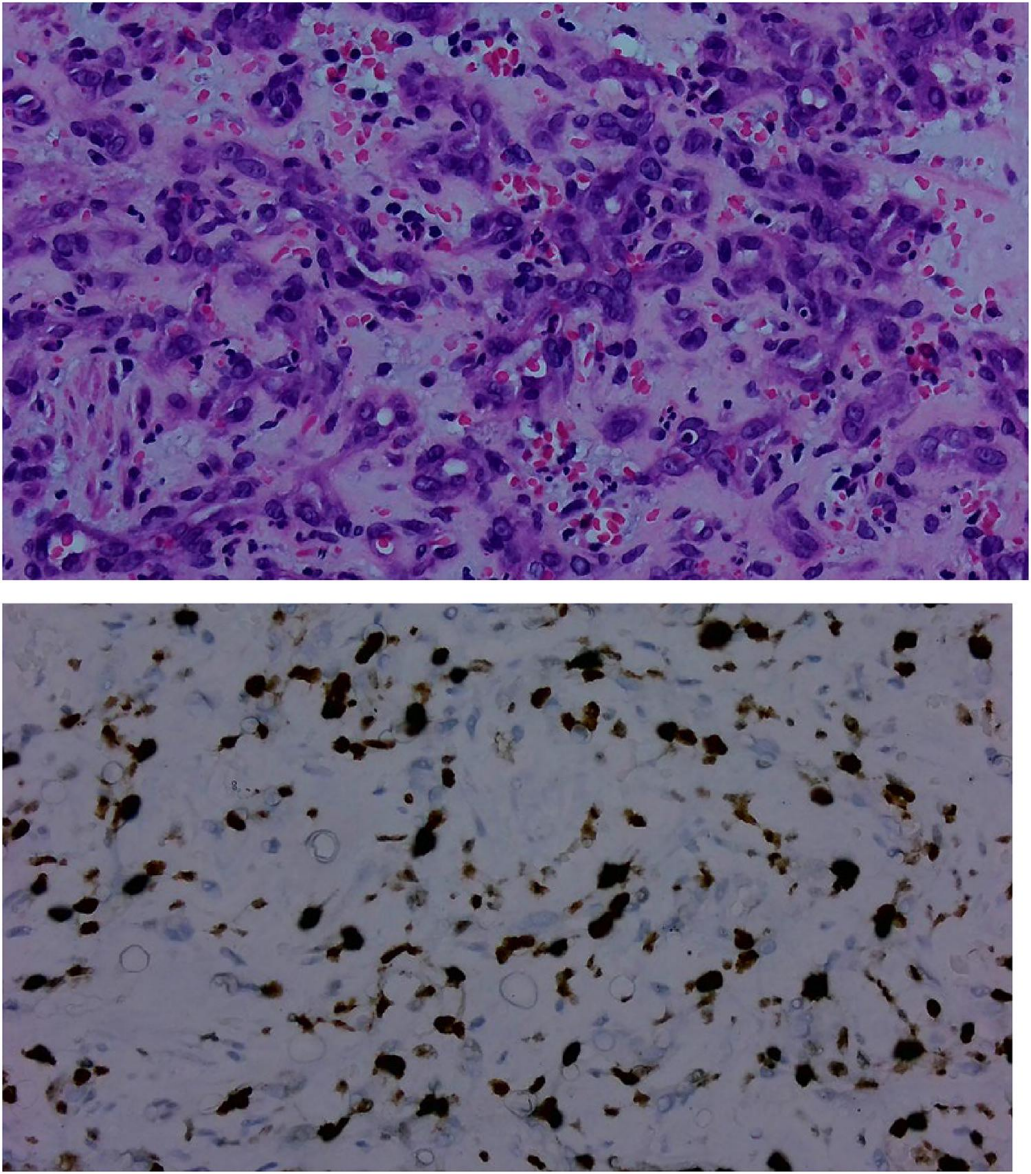
Pathology slides. Photomicrograph of histopathological specimen showing spindle-shaped tumor cells lining split-like capillaries, with pleomorphic vesicular nuclei. Immunohistochemical staining shows strong expression of CD34 and CD31.

The patient underwent endoscopic endonasal excision of the mass without any postoperative complications. Preoperative embolization was not performed. The excised mass was subjected to histopathological examination. Based on the histopathological findings (Figure 2), the final postoperative diagnosis of kaposiform hemangioendothelioma was confirmed. Immunohistochemical analysis revealed positivity for CD34 and CD31. After 5 months of follow-up, there was no evidence of recurrence.

## Discussion

Kaposiform hemangioendothelioma is a rare vascular tumor of endothelial origin, with an estimated incidence of 0.0091% (6). It occurs predominantly in infants and young children, although cases in adults have also been reported (5). Our patient was a 59-year-old woman who presented with a several-month history of right-sided nasal obstruction and epistaxis. Similarly, Hardisson et al. reported the occurrence of this tumor in an adult male who presented with a reddish nodule in the external auditory canal for 1 week (7). The tumor is often associated with the Kasabach–Merritt phenomenon (KMP), which is characterized by thrombocytopenia, hemorrhage, and microangiopathic hemolysis; however, this association is considered a poor prognostic indicator. Lyons et al. reported that approximately 44% of cases of kaposiform hemangioendothelioma did not exhibit the Kasabach–Merritt phenomenon (2). In contrast, Lalaji et al. reported an association between tumors of the right ear and mastoid bone and the Kasabach–Merritt phenomenon (1).

Approximately 75% of lesions present as cutaneous lesions involving the trunk and limbs, followed by the retroperitoneum (18%). Among all sites, the head and neck region is the least commonly affected, with reported locations including the face, external auditory canal, mandible, oropharynx, ethmoid sinus, and internal auditory canal (8). Our patient presented with a several-month history of right-sided nasal obstruction and epistaxis. Very few cases of kaposiform hemangioendothelioma of the nose have been reported to date. A literature review of these cases is summarized in Table 1 (9–11). The tumor is clinically characterized by locally aggressive behavior and a lack of spontaneous regression. It is classified as having intermediate malignant potential by the World Health Organization (5).

**Table 1:** Literature review of previous cases (located in the nose).

Authors	Age	Presenting symptom	Treatment method (surgical technique or medications)	Follow-up duration	Diagnostic images	Recurrence of disease
Wong et al. (9)	46 years/woman	Recurrent left-sided epistaxis and hyposmia	Medial maxillectomy, anterior and posterior ethmoidectomy, and *en bloc* excision of the tumor via a lateral rhinotomy approach	1 year	MRI of the PNS revealed a homogeneous sinonasal mass lesion (3.2 cm×2 cm), centered in the left middle and upper nasal cavity, with involvement of the anterior and posterior ethmoid	No
Qurratulain Chundriger et al. (10)	10 years /girl	Not mentioned	Not mentioned	120 months	Not provided	No
van Zijl (11)	2 years /boy	Purpuric lesion of the nose	Only biopsy and medical treatment (sirolimus) for 4 years	4 years	MRI scan revealed a subcutaneous infiltration extending into the nasal cavity and anterior ethmoid sinus with bright heterogeneous enhancement	No
Chin-Ho Lee et al. (21)	4 months/girl	Swelling of the right cheek	Not mentioned, only biopsy was mentioned	Not mentioned	CT showed an isodense to hyperdense mass mainly involving the right maxillary sinus, with associated bone destruction and extension into the right middle cranial fossa and right orbit. MRI revealed the mass to be hypointense on fat-suppressed T2-weighted and slightly hypointense on T1-weighted, reticular enhancement with contrast	Not mentioned

MRI, magnetic resonance imaging; CT, computed tomography.

The diagnosis is usually based on histopathological and immunohistochemical findings. Histopathologically, the tumor shows features similar to those of benign capillary hemangiomas, including vessels with round or oval lumens, and those of Kaposi's sarcoma, such as the presence of spindled neoplastic endothelial cells and intracellular and extracellular hyaline globules. The only feature that distinguishes it from the above two entities is the absence of periodic acid–Schiff-positive globules and the presence of well-formed spindle cell fascicles (1). Immunohistochemically, several immature endothelial cells express cluster of differentiation markers (CD31 and CD34) (12).

CT and magnetic resonance imaging (MRI) are radiologically useful. Enhanced images are acquired preoperatively. For postoperative follow-up, MRI is preferred. In cases where the tumor is large and highly vascular, CT or MRI angiography may be required, and preoperative embolization can be considered. In the present case, preoperative embolization was not performed because of the location and size of the mass. Radiologically, the tumor (KHE) appears as an expansile osteolytic lesion with irregular margins, often penetrating adjacent soft tissues (8). On MRI, the lesion typically demonstrates a well-enhanced soft tissue component that shows hyperintensity on T2-weighted images and hypointensity on T1-weighted images (13). Using CT and MRI, Ibarra et al. described the entity as a large, well-defined, expansile osteolytic lesion involving the temporal bone along with the presence of hemorrhagic foci (14). In another study by Panow et al., kaposiform hemangioendotheliomas of the mandible exhibited infiltrative mass formation on MRI and spiculated features on CT imaging (15). The differential diagnosis of sinonasal tumors can be classified into three categories: non-neoplastic masses like allergic polyp, inflammatory polyp, rhinoscleroma, and rhinosporodiosis; benign neoplastic masses like hemangioma, inverted papilloma, mucocele, and angiofibroma; and malignant neoplastic masses like squamous cell carcinoma and adenocarcinoma (20).

Surgical excision remains the treatment of choice for kaposiform hemangioendothelioma and may occasionally be combined with radiotherapy. In the present case, the patient underwent excision of the mass via an endoscopic endonasal approach. In addition, several medications, such as interferons, prednisolone, and vincristine, have been used to increase the effectiveness of treatment (16, 17). A case report of an infant who was diagnosed with kaposiform hemangioendothelioma of the chest showed significant improvement in both tumor size and coagulopathy following treatment with sirolimus (19).

In refractory cases, second-line treatment with vincristine may be administered either as monotherapy or in combination with cyclophosphamide, actinomycin D, and methotrexate (17).

The prognosis of the tumor usually depends on its size, anatomical location, and extent. Close observation for recurrence during follow-up is recommended. Multivariate analysis results have identified age of onset and lesion size as risk factors for the KMP, a severe complication associated with KHE (18).

In a case of KHE in the external auditory canal by Hardisson et al., recurrence occurred within 1 month of surgery and was later treated with radiotherapy (7). Among the reported cases involving the nose (Table 1), no recurrences were observed in any of the three cases. However, in our case, no recurrence was noted after 5 months and 1 year of follow-up.

## Conclusion

Kaposiform hemangioendothelioma is a locally aggressive tumor with a rare risk of metastasis. Awareness of this condition should be increased to enhance the diagnosis of such cases in adult patients.

## Data Availability

The original contributions presented in the study are included in the article/Supplementary Material; further inquiries can be directed to the corresponding author.

## References

[1] Lalaji TA Haller JO Burgess RJ. A case of head and neck kaposiform hemangioendothelioma simulating a malignancy on imaging. Pediatr Radiol. (2001) 31:876–78. 10.1007/s002470100009 11727024

[2] Lyons LL North PE Mac-Moune Lai F Stoler MH Folpe AL Weiss SW. Kaposiform hemangioendothelioma: a study of 33 cases emphasizing its pathologic, immunophenotypic and biologic uniqueness from juvenile hemangioma. Am J Surg Pathol. (2004) 28:559–68. 10.1097/00000478-200405000-00001 15105642

[3] Al-Rashid RA. Cyclophosphamide and radiation therapy in the treatment of hemangioendothelioma with disseminated intravascular clotting. Cancer. (1971) 27:364–8. 10.1002/1097-0142(197102)27:2<364::aid-cncr2820270219>3.0.co;2-v 5541951

[4] Zukerberg LR Nickoloff BJ Weiss SW. Kaposiform hemangioendothelioma of infancy and childhood: an aggressive neoplasm associated with Kasabach–Merritt syndrome and lymphangiomatosis. Am J Surg Pathol. (1993) 17:321–8. 10.1097/00000478-199304000-00001 8494101

[5] Tsang WY. Kaposiform hemangioendothelioma. In: Fletcher C Unni K Mertens F, editors. Pathology and Genetics of Tumors of Soft Tissue and Bone. Lyon, France: IARC Press (2002). p. 163–4.

[6] Croteau SE Liang MG Kozakewich HP Alomari AI Fishman SJ Mulliken JB Kaposiform hemangioendothelioma: atypical features and risks of Kasabach–Merritt phenomenon in 107 referrals. J Pediatr. (2013) 162:142–7. 10.1016/j.jpeds.2012.06.044 22871490 PMC3494787

[7] Hardisson D Prim MP Diego J Patron M Escribano A Rabanal I. Kaposiform hemangioendothelioma of the external auditory canal in an adult male. Head Neck. (2002) 24:614–7. 10.1002/hed.10074 12112561

[8] Chang JM Kwon BJ Han MH Kang HS Chang KH. Kaposiform hemangioendothelioma arising from the internal auditory canal. Am J Neuroradiol. (2006) 27:931–3. https://pmc.ncbi.nlm.nih.gov/articles/PMC8133982/16611794 PMC8133982

[9] Wong BLK Dwivedi RC Masterson L Riffat F Marker A Jani P. Kaposiform hemangioendothelioma of paranasal sinus. Laryngoscope. (2014) 124(9):2103–6. 10.1002/lary.24669 24619771

[10] Chundriger Q Tariq MU Abdul-Ghafar J Ahmed A Din NU. Kaposiform hemangioendothelioma: clinicopathological characteristics of 8 cases of a rare vascular tumor and review of literature. Diagn Pathol. (2021) 16:23. 10.1186/s13000-021-01080-9 33722245 PMC7962213

[11] van Zijl FVWJ de Laat PCJ Verdijk RM Nagtegaal AP Datema FR. Aggressive vascular tumor mimicking posttraumatic hematoma: a case report of kaposiform hemangioendothelioma on the nose. JAAD Case Rep. (2022) 26:45–8. 10.1016/j.jdcr.2022.05.041 35865722 PMC9294492

[12] Birchler MT Schmid S Holzmann D Stallmach T Gysin C. Kaposiform hemangioendothelioma arising in the ethmoid sinus of an 8-year-old girl with severe epistaxis. Head Neck. (2006) 28:761–4. 10.1002/hed.20414 16721737

[13] Chen YJ Wang CK Tien YC Hsieh T-J. MRI of multifocal kaposiform haemangioendothelioma without Kasabach–Merritt phenomenon. Br J Radiol. (2009) 82:e51–4. 10.1259/bjr/16482217 19211904

[14] Ibarra RA Kesava P Hallet KK Bogaev C. Hemangioendothelioma of the temporal bone with radiologic findings resembling hemangioma. AJNR Am J Neuroradiol. (2001) 22:755–58. https://pmc.ncbi.nlm.nih.gov/articles/PMC7976014/11290494 PMC7976014

[15] Panow C Berger C Willi U Valavanis A Martin E. MRI and CT of a hemangioma of the mandible in Kasabach–Merritt syndrome. Neuroradiology. (2000) 42:215–17. 10.1007/s002340050050 10772147

[16] Chung MT Chen CH Chiu CH Yang CP Hsueh C Jaing TH. Successful nonoperative therapy for kaposiform hemangioendothelioma involving the neck: report of 1 case. Otolaryngol Head Neck Surg. (2003) 129:605–07. 10.1016/S0194-59980300725-3 14595290

[17] Haisley-Royster C Enjolras O Frieden IJ Garzon M Lee M de Laat PCJ Kasabach–Merritt phenomenon: a retrospective study of treatment with vincristine. J Pediatr Hematol Oncol. (2002) 24:459–62. 10.1097/00043426-200208000-00010 12218593

[18] Zhou J Lan Y Qiu T Gong X Zhang Z He C Impact of age and tumor size on the development of the Kasabach–Merritt phenomenon in patients with kaposiform hemangioendothelioma: a retrospective cohort study. Precis Clin Med. (2023) 6(2):pbad008. 10.1093/pcmedi/pbad008 37305527 PMC10249050

[19] Nakamura S Ozeki M Hayashi D Yasue S Endo S Ohnishi H. Sirolimus monotherapy for Kasabach–Merritt phenomenon in a neonate; case report. Int J Surg Case Rep. (2024) 117:109497. 10.1016/j.ijscr.2024.109497 38518465 PMC10972789

[20] Lathi A Syed MM Kalakoti P Qutub D Kishve SP. Clinico-pathological profile of sinonasal masses: a study from a tertiary care hospital of India. Acta Otorhinolaryngol Ital. (2011) 31(6):372–7. https://pmc.ncbi.nlm.nih.gov/articles/PMC3272868/22323848 PMC3272868

[21] Lee CH Jaw TS Yang SF Wu DK. Kaposiform hemangioendothelioma arising from the maxillary sinus: a case report. Kaohsiung J Med Sci. (2010) 26(3):154–7. 10.1016/S1607-551X(10)70023-1 20227656 PMC11916336

